# Contaminants in the cow's milk we consume? Pasteurization and other technologies in the elimination of contaminants

**DOI:** 10.12688/f1000research.108779.1

**Published:** 2022-01-25

**Authors:** Micaela Belen Calahorrano-Moreno, Jonathan Jerry Ordoñez-Bailon, Ricardo José Baquerizo-Crespo, Alex Alberto Dueñas-Rivadeneira, Maria Conceição B. S. M. Montenegro, Joan Manuel Rodríguez-Díaz

**Affiliations:** 1 Departamento de Procesos Químicos, Facultad de Ciencias Matemáticas, Físicas y Químicas, Universidad Técnica de Manabí, Portoviejo, Manabí, 130104, Ecuador; 2 Departamento de Procesos Agroindustriales, Facultad de Ciencias Zootécnicas, Universidad Técnica de Manabí, Portoviejo, Manabí, 130104, Ecuador; 3 REQUIMTE/Departamento de Ciências Químicas, Faculdade de Farmácia, Universidade do Porto, Porto, Porto, 4050-313, Portugal; 4 Laboratorio de Análisis Químicos y Biotecnológicos, Instituto de Investigación, Universidad Técnica de Manabí, Portoviejo, Manabí, 130104, Ecuador

**Keywords:** human health; chemical contaminant; microbiological contaminant; alternative; technology; food safety

## Abstract

Cow's milk is currently the most consumed product worldwide. However, due to various direct and indirect contamination sources, different chemical and microbiological contaminants have been found in cow's milk. This review details the main contaminants found in cow's milk, referring to the sources of contamination and their impact on human health. A comparative approach highlights the poor efficacy and effects of the pasteurization process with other methods used in the treatment of cow's milk. Despite pasteurization and related techniques being the most widely applied to date, they have not demonstrated efficacy in eliminating contaminants. New technologies have appeared as alternative treatments to pasteurization. However, in addition to causing physicochemical changes in the raw material, their efficacy is not total in eliminating chemical contaminants, suggesting the need for new research to find a solution that contributes to improving food safety.

## 1. Introduction

Milk is a fluid secreted by the female of the mammalian species and fulfills the nutritional requirements of the neonate, for instance: (i) the energetic part (provided by lipids, lactose, and in excess by proteins), essential amino acids, and (ii) amino groups necessary for the biosynthesis of non-essential amino acids (provided by proteins), essential fatty acids, vitamins, inorganic elements, and water. ^1^

Global milk production has increased by about 20% in the last decade, from 694 million tons in 2008 ^2^ to 843 million tons in 2018. ^3^ As a result, bovine milk is the most consumed food product representing about 48% of the total milk consumed globally, the European Union (EU), Australia, and New Zealand being the most important producers, followed by the United States and India. ^4^

Collection and processing expose milk to different contaminants, mainly pesticide residues, metals, mycotoxins, hormones, and others reaching the cow through feeding or drug administration by producers. ^5^ Thus, milk can contain hazardous materials, of either biological or chemical origin.

Although pasteurization has been an efficient antimicrobial method and has contributed to reducing many diseases, several infectious episodes associated with pasteurized milk have continued to occur, mainly when raw milk has an exaggerated population of microorganisms that increase the margin of survival and by post pasteurization contamination. ^6^ The biggest problem of pathogens in pasteurized milk is that they persist without causing any organoleptic alteration, increasing sanitary risk since the consumer cannot suspect their presence, showing that pasteurization has some drawbacks in treating pathogens. ^7^

As population and industrial growth increased, new contaminants appeared, and with this, contamination of cow's milk also increased not only by compounds of biological origin but also by compounds of chemical origin, as mentioned above. ^8^ However, pasteurization has remained the only established treatment, even though it is only effective for eliminating most biological and non-chemical compounds. ^9^ In contrast, the literature mentions very few alternative treatments to treat chemical contaminants in cow's milk, leading to a critical analysis of their application to ensure sufficient quality in the milk consumed. Given this evidence, the bibliographic review here aims to identify the different types of contaminants in raw/pasteurized cow's milk and analyze the application of alternative processes for the elimination or degradation of contaminants.

## 2. Contaminants present in cow's milk

There are several hazards of contamination of cow's milk, ranging from biological to chemical compounds. The risk of biological contamination of cow's milk derives mainly from cattle milking due to the exposure of udders to the environment, equipment, storage, dirty pipes, and others. ^10^ Chemical contamination of cow's milk comes from several sources: application of agrochemicals, ^11^ use of legal or illegal veterinary products, ^12^ feed and forages contaminated with natural toxins, ^13^ or through the improper use of chemicals during milk production, processing and packaging stages. ^14^

**Figure 1.**
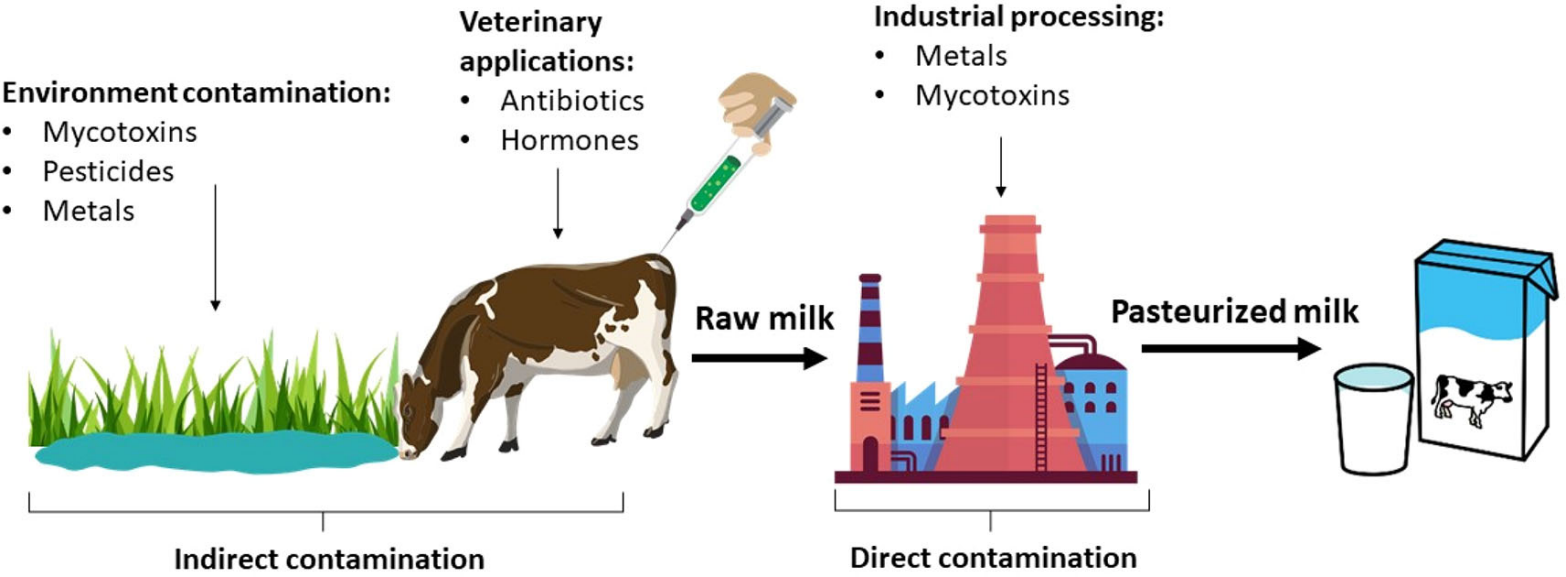
Sources of contamination of bovine milk.

Figure 1 shows the direct and indirect pathways for contaminants entry into bovine milk.

Indirect contamination is associated with the ingestion of contaminants both from the environment and from substances of veterinary use. The most common environmental contaminants are mycotoxins, pesticides, and metals consumed by cattle through feed, forages, and water. In addition, antibiotics and hormones are administered to the cow orally, by injection, or as intramammary infusions to treat diseases, promote animal growth and increase milk production. ^5^ On the other hand, direct contamination occurs during milk processing from milking, handling, storage and even pasteurization. During the industrialization process, milk comes into contact with metals, residues of cleaning products, mycotoxins, among others.

**Figure 2.**
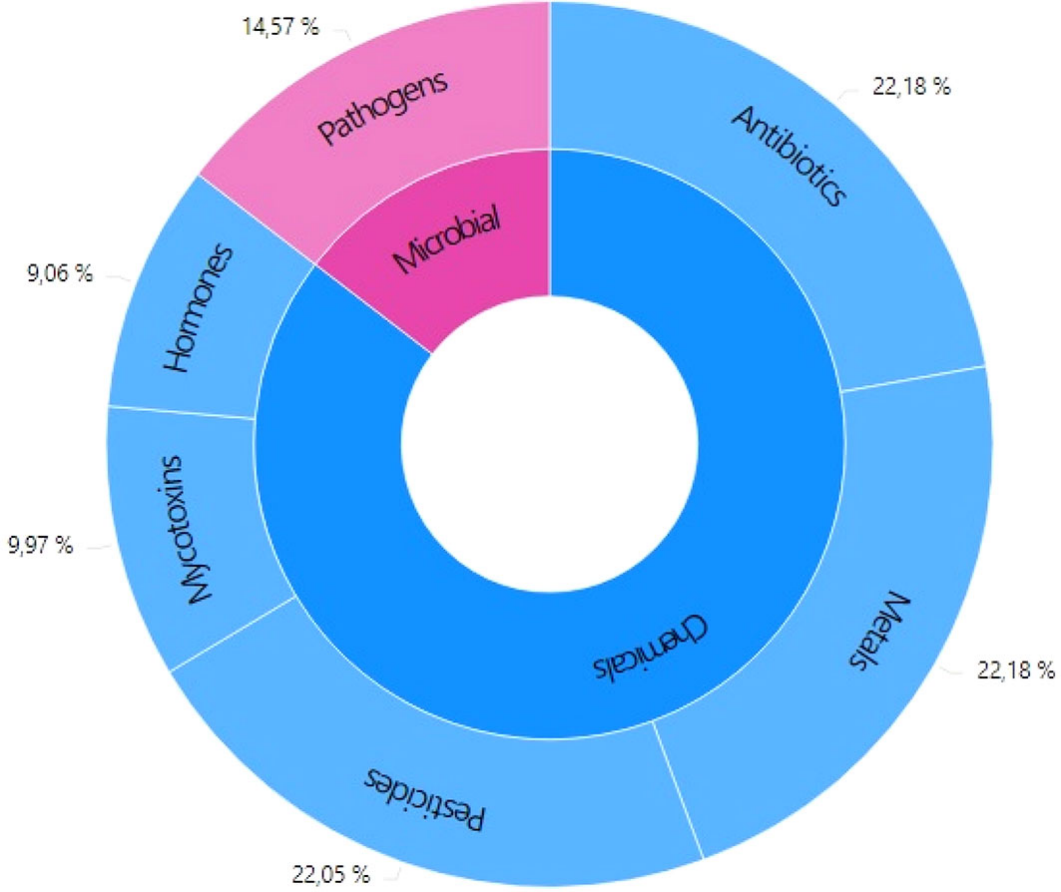
Distribution of literature related to contaminants in bovine milk between 2010-2021.

For better analysis and understanding, the classification of contaminants according to the origin is microbial contaminants and chemical contaminants ( Figure 2).

About 14.57% of the literature reports contamination of cow's milk by pathogenic microorganisms. Although the objective of the pasteurization process is the elimination of these microorganisms, there is evidence of their presence in pasteurized milk, which will be presented later. Although pathogenic microorganisms are considered the main hazard that threatens the safety of milk, they do not represent the highest percentage of reported cases. The contaminants that have been more reported in the literature are of chemical origin ( Figure 2). Among chemical contaminants, metals, pesticides, and antibiotics stand out. Among chemical contaminants, the most reported are heavy metals (22.18%), pesticides (22.05%), and antibiotics (22.18%); due to bad practices in agriculture and cattle. Although reports of mycotoxins in milk are relatively low (9.97%), they are of great importance due to the increase in reported cases of contamination with Aflatoxin M1 (AFM1). The International Agency for Research on Cancer has classified AFM1 as a carcinogenic substance. ^15^ This means that the food safety of milk is at risk, as any of these compounds compromise the health of the final consumer. Below is a detailed classification of the different types of contaminants present in both raw and pasteurized milk and the negative effects they have on consumer health.

### 2.1 Microbial contaminants

The presence of several pathogenic microorganisms has been reported in raw and pasteurized cow's milk ( Table 1). Microbial contamination of raw milk can be due to diseases such as mastitis, improper handling on production farms, milking equipment, water sources, and feeding of cattle, utensils, and equipment used for milk storage on the farm or during transport. ^16^ Likewise, poor hygienic practices within the dairy industry can lead to the formation of biofilms on the sprinklers of cooling systems, pipes, cooling tanks, storage, and transport tanks. The contact of pasteurized milk with these surfaces increases the risk of contamination with pathogenic microorganisms, posing a danger to the consumer and the quality of the product. ^17^

**Table 1.** Pathogens in bovine milk reported in literature.

Pathogens	Type of milk	Reference
*Mycobacterium*	Raw	^18^^,^^19^
*Pseudomonas*	Raw	^20^^,^^21^^,^^22^^,^^23^^,^^24^^,^^25^^,^^16^^,^^26^^,^^27^^,^^28^^,^^29^
	Pasteurized	^16^
*Hafnia*	Raw	^25^^,^^23^^,^^21^
*Serratia*	Raw	^22^^,^^25^^,^^24^^,^^21^
*Klebsiella*	Raw	^30^
	Pasteurized	^30^
*Citrobacter*	Raw	^25^
*Escherichia*	Raw	^16^^,^^30^^,^^31^
	Pasteurized	^16^^,^^30^
*Staphylococcus*	Raw	^32^^,^^27^^,^^28^^,^^29^
*Bacillus*	Raw	^27^^,^^28^^,^^20^^,^^21^
*Lactococcus*	Raw	^32^^,^^26^^,^^21^^,^^27^^,^^28^^,^^20^
*Corynebacterium*	Raw	^28^
*Streptococcus*	Raw	^27^^,^^28^
*Enterobacter*	Raw	^27^^,^^28^^,^^25^
*Mycoplasma*	Raw	^27^
*Enterococcus*	Raw	^27^^,^^28^^,^^21^^,^^32^
*Acinetobacter*	Raw	^27^^,^^28^^,^^20^^,^^29^^,^^26^^,^^33^^,^^32^
*Sneathia*	Raw	^27^
*Kocuria*	Raw	^27^^,^^28^^,^^32^
*Neisseria*	Raw	^27^
*Fusobacterium*	Raw	^27^
*Macrococcus*	Raw	^27^
*Trueperella*	Raw	^27^
*Halomonas*	Raw	^27^
*Micrococcus*	Raw	^27^
*Enhydrobacter*	Raw	^27^
*Psychrobacter*	Raw	^27^^,^^28^
*Campylobacter*	Raw	^34^^,^^31^^,^^35^
*Brachybacterium*	Raw	^28^
*Dermacoccus*	Raw	^28^
*Leucobacter*	Raw	^28^
*Microbacterium*	Raw	^28^^,^^20^
*Aerococcus*	Raw	^28^
*Lactobacillus*	Raw	^28^^,^^33^
*Ochrobactrum*	Raw	^28^
*Pantoea*	Raw	^28^
*Paracoccus*	Raw	^28^
*Sphingomonas*	Raw	^28^
*Deinococcus*	Raw	^28^
*Aspergillus*	Raw	^28^
*Cladosporium*	Raw	^28^
*Eurotium*	Raw	^28^
*Penicillium*	Raw	^28^
*Wallemia*	Raw	^28^
*Listeria*	Raw	^31^^,^^36^
*Yersinia*	Raw	^31^^,^^36^
*Salmonella*	Raw	^30^^,^^16^
	Pasteurized	^30^^,^^16^
*Vibrio*	Raw	^30^
	Pasteurized	^30^
*Stenotrophomonas*	Raw	^33^^,^^32^
*Chryseobacterium*	Raw	^33^
*Paenibacillus*	Raw	^20^^,^^21^
*Coliforms*	Pasteurized	^37^

According to Table 1, Most cases of contamination are recorded in raw milk due to inadequate milking, processing, storage, and transport conditions. On the other hand, although few studies report the presence of microorganisms in pasteurized milk, it is doubtful that it is an efficient process for their elimination. The main types of microorganisms present in milk are bacteria, yeasts, and molds, which represent the different types of microorganisms present in cow's milk. The presence of *Corynebacteria*, *Staphylococcus*, *Streptococcus*, *Bacillu*s, and *Micrococcus* species has been evidenced in the teat of dairy cattle. ^38^^,^^39^ These microorganisms have also been identified in cow's milk, ^27^^,^^28^^,^^40^^,^^41^ demonstrating that during milking, milk can become contaminated by contact with the cow's teat under unhygienic conditions. On the other hand, as a result of mastitis, *Staphylococcus* and *Streptococcus* species have been identified in bovine milk samples, ^42^^,^^43^ with *Staphylococcus aureus* being the main cause of mastitis. ^43^ The presence of *Enterobacteriaceae*, *Pseudomonas* spp., *Staphylococcus* spp., and lactic acid bacteria has been identified in the equipment used for milking. ^17^ It is evident that the conditions under which milk is obtained on farms are not the most adequate because these different microorganisms are found in cow's milk. ^33^^,^^42^^,^^44^^,^^45^

Consumption of milk contaminated by pathogenic microorganisms such as *Campylobacter*, *Salmonella*, *Yersinia*, *E. coli*, *Listeria*, and *S. aureus* can cause muscle and stomach pain, gastrointestinal diseases with diarrhea, fever, and nausea. ^31^ These microorganisms are commonly found in the intestinal flora or in the udder of cows, thus facilitating milk contamination. ^31^ In addition, *Campylobacter* spp. and *E. Coli* O157:H7 are capable of producing Guillain-Barrés syndrome and hemolytic uremic syndrome, respectively. ^46^

### 2.2 Chemical contaminants

For a more detailed analysis, the chemical contaminants found in cow's milk have been classified into five groups: pesticides, metals, antibiotics, mycotoxins, and hormones ( Table 2).

**Table 2.** Chemical contaminants in bovine milk reported in literature.

Compounds		Type of milk	MRL ^a^ (μg/kg)	MRL ^b^ (μg/kg)	Reference
Pesticides	Hexachlorocyclohexane (HCH)	Raw	-	-	^11^^,^^47^^,^^48^^,^^49^^,^^50^^,^^51^
		Pasteurized	-	-	^52^^,^^53^^,^^51^
		UHT	-	-	^47^
	Butachlor	Raw	-	-	^11^
		Pasteurized	-	-	^54^
	Cyhalothrin	Raw	30	50	^11^^,^^55^^,^^56^
		Pasteurized	-	-	^54^
	Cypermethrin	Raw	100	20	^11^^,^^56^^,^^57^
		Pasteurized	-	-	^54^^,^^58^^,^^59^
	Fenvalerate	Raw	-	40	^11^
		Pasteurized	-	-	^59^
	Deltamethrin	Raw	30	20	^11^^,^^55^^,^^56^^,^^57^^,^^55^
		Pasteurized	-	-	^60^
	Malathion	Raw	-	-	^11^^,^^61^^,^^62^
	Chlorpyrifos	Raw	-	-	^11^^,^^55^^,^^56^^,^^61^^,^^55^^,^^60^
		Pasteurized	-	-	^54^^,^^58^^,^^59^^,^^60^
		UHT	-	-	^60^
	Carbofuran	Raw	-	-	^55^^,^^62^
	Permethrin	Raw	-	50	^56^^,^^57^
		Pasteurized	-	-	^54^^,^^58^^,^^59^
	Profenophos	Raw	-	-	^11^^,^^60^
		Pasteurized	-	-	^54^^,^^60^
		UHT	-	-	^60^
	Ethion	Raw	-	-	^11^
		Pasteurized	-	-	^54^^,^^63^
	Dichloro diphenyl trichloroethane (DDT)	Raw	-	-	^11^^,^^64^^,^^65^^,^^47^^,^^66^^,^^48^^,^^50^^,^^51^
		Pasteurized	-	-	^64^^,^^52^^,^^54^^,^^67^^,^^51^
		UHT	-	-	^47^
	Dicofol	Pasteurized	-	-	^59^
	Aldrin+Dieldrin	Raw	-	-	^64^^,^^47^^,^^49^
		Pasteurized	-	-	^64^^,^^53^
		UHT	-	-	^47^
	Endrin	Raw	-	-	^68^^,^^69^^,^^49^
		Pasteurized	-	-	^70^^,^^53^^,^^54^^,^^67^
	Fipronil	Raw	-	-	^11^^,^^65^^,^^60^
		Pasteurized	-	-	^54^^,^^60^
	Hexaflumuron	Raw	-	-	^65^
	Teflubenzuron	Raw	-	-	^65^
	Diflufenican	Raw	-	-	^65^
	Piperophos	Raw	-	-	^65^
	Dimethoate	Raw	-	-	^60^^,^^62^
		Pasteurized	-	-	^60^
	Atrazine	Pasteurized	-	-	^58^^,^^59^
	Diazinon	Raw	-	20	^62^^,^^60^
		Pasteurized	-	-	^58^^,^^59^^,^^60^
		UHT	-	-	^60^
	Lindane	Raw	-	-	^64^^,^^51^
		Pasteurized	-	-	^64^^,^^51^
	Endosulfane	Raw	-	-	^11^^,^^65^^,^^47^^,^^68^^,^^48^^,^^71^^,^^49^^,^^51^^,^^56^
		Pasteurized	-	-	^52^^,^^70^^,^^53^^,^^54^^,^^67^
		UHT	-	-	^47^
	Hexachlorobenzene	Raw	-	-	^72^
		Pasteurized	-	-	^70^^,^^58^^,^^59^
	Heptachlor epoxide	Raw	-	-	^65^^,^^47^^,^^73^^,^^68^
		Pasteurized	-	-	^59^
		UHT	-	-	^47^^,^^73^
	Heptachlor	Raw	-	-	^68^^,^^69^^,^^51^
		Pasteurized	-	-	^73^^,^^52^^,^^70^^,^^51^
		UHT	-	-	^73^
	Chlordane	Pasteurized	-	-	^52^^,^^53^^,^^67^
	Methoxychlor	Raw	-	-	^47^^,^^69^
		Pasteurized	-	-	^54^
		UHT	-	-	^47^
	Azoxystrobin	Pasteurized	-	-	^74^
	Chlorantranilliprole	Pasteurized	-	-	^74^
	Flubendiamide	Pasteurized	-	-	^74^
	Imidacloprid	Raw	-	-	^55^
		Pasteurized	-	-	^74^
	Lufenuron	Pasteurized	-	-	^74^
	Metalaxyl	Pasteurized	-	-	^74^
	Novaluron	Pasteurized	-	-	^74^
	Uniconazol	Pasteurized	-	-	^74^
	Monuron	Pasteurized	-	-	^75^
	Methabenzthiazuron	Pasteurized	-	-	^75^
	Buturon	Pasteurized	-	-	^75^
	Linuron	Pasteurized	-	-	^75^
	Aziprotryne	Pasteurized	-	-	^75^
	Bitertanol	Pasteurized	-	-	^75^
	Clofentezine	Pasteurized	-	-	^75^
	Methyl Parathion	Raw	-	-	^62^^,^^76^
Metals	Cadmium	Raw	-	-	^77^^,^^78^^,^^79^^,^^80^^,^^81^^,^^82^^,^^83^^,^^84^^,^^85^^,^^86^
		Pasteurized	-	-	^87^^,^^77^^,^^88^
		UHT	-	-	^89^^,^^90^
	Lead	Raw	-	-	^77^^,^^91^^,^^78^^,^^79^^,^^80^^,^^81^^,^^82^^,^^83^^,^^84^^,^^92^^,^^85^^,^^86^
		Pasteurized	-	-	^87^^,^^77^^,^^93^^,^^88^
		UHT	-	-	^92^^,^^94^^,^^89^^,^^90^
	Copper	Raw	-	-	^77^^,^^79^^,^^80^^,^^81^^,^^82^^,^^84^^,^^92^^,^^89^^,^^88^^,^^85^^,^^86^
		Pasteurized	-	-	^87^^,^^77^^,^^93^^,^^88^
		UHT	-	-	^92^^,^^94^^,^^89^^,^^90^
	Zinc	Raw	-	-	^77^^,^^95^^,^^80^^,^^81^^,^^82^^,^^88^^,^^85^^,^^96^^,^^86^
		Pasteurized	-	-	^77^^,^^93^^,^^88^^,^^96^
		UHT	-	-	^94^^,^^89^^,^^90^
	Selenium	Raw	-	-	^82^^,^^85^^,^^96^^,^^86^
		Pasteurized	-	-	^96^
		UHT	-	-	^94^
	Chromium	Raw	-	-	^77^^,^^91^^,^^88^^,^^85^^,^^96^^,^^86^
		Pasteurized	-	-	^77^^,^^93^^,^^88^^,^^96^
		UHT	-	-	^90^
	Nickel	Raw	-	-	^77^^,^^91^^,^^79^^,^^97^^,^^88^^,^^85^^,^^86^
		Pasteurized	-	-	^77^^,^^93^^,^^88^
		UHT	-	-	^94^^,^^89^
	Iron	Raw	-	-	^80^^,^^82^^,^^89^^,^^88^^,^^85^^,^^96^^,^^86^
		Pasteurized	-	-	^93^^,^^88^^,^^96^
		UHT	-	-	^94^^,^^89^^,^^90^
	Arsenic	Raw	-	-	^98^^,^^91^^,^^83^^,^^84^^,^^97^^,^^88^^,^^85^^,^^96^^,^^86^
		Pasteurized	-	-	^88^^,^^96^
	Magnesium	Raw	-	-	^95^^,^^82^^,^^83^^,^^88^^,^^85^^,^^86^
		Pasteurized	-	-	^93^^,^^88^
		UHT	-	-	^90^
	Manganese	Raw	-	-	^82^^,^^89^^,^^88^^,^^85^^,^^86^
		Pasteurized	-	-	^93^^,^^88^
		UHT	-	-	^89^^,^^90^
	Aluminum	Raw	-	-	^98^^,^^91^^,^^85^^,^^96^^,^^86^
		Pasteurized	-	-	^96^
	Molybdenum	Raw	-	-	^98^^,^^86^
	Mercury	Raw	-	-	^91^^,^^84^^,^^99^^,^^97^^,^^88^^,^^86^
		Pasteurized	-	-	^88^
		UHT	-	-	^94^
	Tin	Raw	-	-	^97^^,^^85^^,^^86^
	Cobalt	Raw	-	-	^77^^,^^79^^,^^89^^,^^88^^,^^86^
		Pasteurized	-	-	^77^^,^^88^
		UHT	-	-	^94^^,^^89^
Antibiotics	Oxytetracycline	Raw	100	100	^100^^,^^101^^,^^102^^,^^103^^,^^104^^,^^105^
		Pasteurized	-	-	^106^^,^^58^^,^^107^^,^^102^^,^^108^^,^^105^^,^^109^
		UHT	-	-	^102^^,^^108^
	Lincomycin	Raw	150	150	^100^^,^^101^^,^^110^
		Pasteurized	-	-	^111^^,^^112^
		UHT	-	-	^111^
	Quinolone	Raw	-	-	^104^^,^^113^
		Pasteurized	-	-	^111^^,^^114^^,^^113^
		UHT	-	-	^111^^,^^114^
	Tetracycline	Raw	100	100	^102^^,^^103^^,^^115^^,^^116^^,^^104^^,^^105^^,^^110^
		Pasteurized	-	-	^111^^,^^114^^,^^117^^,^^107^^,^^102^^,^^108^^,^^109^
		UHT	-	-	^111^^,^^117^^,^^102^^,^^108^
	Doxycicline	Raw	-	-	^103^^,^^104^
		Pasteurized	-	-	^106^
		UHT	-	-	^108^
	Penicillin G	Raw	-	-	^101^^,^^118^^,^^119^^,^^120^^,^^121^^,^^122^
		Pasteurized	-	-	^106^^,^^109^
	Trimethoprim	Raw	-	50	^123^
	Amoxicillin	Raw	4	4	^124^^,^^119^^,^^120^^,^^121^^,^^122^
		Pasteurized	-	-	^58^^,^^109^
	Cefalexin	Raw	-	100	^120^^,^^125^
	Cephapirin	Raw	-	60	^101^^,^^120^
	Fleroxacin	Raw	-	-	^126^
	Chlortetracycline	Raw	100	100	^102^^,^^104^
		Pasteurized	-	-	^102^
		UHT	-	-	^102^^,^^108^
	Enrofloxacin	Raw	-	100	^126^^,^^127^^,^^115^^,^^116^^,^^128^^,^^105^^,^^113^^,^^129^^,^^119^^,^^120^^,^^122^^,^^110^
		Pasteurized	-	-	^127^^,^^108^^,^^105^
		UHT	-	-	^108^
	Ciprofloxacin	Raw	-	100	^126^^,^^127^^,^^103^^,^^129^^,^^119^^,^^120^^,^^122^
		Pasteurized	-	-	^127^^,^^108^
		UHT	-	-	^108^
	Lomefloxacin	Raw	-	-	^126^
	Tilmicosin	Pasteurized	-	50	^112^^,^^130^
	Erythromycin A	Pasteurized	-	40	^130^
	Tylosin	Raw	100	50	^103^^,^^116^
		Pasteurized	-	-	^112^^,^^109^
	Spiramycin	Pasteurized	200	200	^112^
	Streptomycin	Raw	200	200	^131^^,^^116^^,^^128^^,^^110^
		Pasteurized	-	-	^111^
		UHT	-	-	^111^
	Gentamicin	Raw	200	100	^131^^,^^116^^,^^128^
		Pasteurized	-	-	^109^
		UHT	-	-	^108^
	Gatifloxacin	Raw	-	-	^127^
		Pasteurized	-	-	^127^
	Ofloxacin	Raw	-	-	^127^
		Pasteurized	-	-	^127^^,^^132^
	Norfloxacin	Raw	-	-	^127^
		Pasteurized	-	-	^127^^,^^108^
		UHT	-	-	^108^
	Sulfamethoxazole	Raw	-	-	^127^^,^^103^^,^^105^^,^^123^
		Pasteurized	-	-	^127^
	Sulfamethazine	Pasteurized	-	-	^127^^,^^58^
		UHT	-	-	^114^
	Sulfadimethoxine	Raw	-	-	^103^
		Pasteurized	-	-	^58^
	Sulfadiazine	Pasteurized	-	-	^133^
	Sulfathioazole	Pasteurized	-	-	^58^
	Ceftiofur	Raw	-	-	^103^
	Sulfonamides	Raw	-	-	^116^^,^^128^^,^^104^
		Pasteurized	-	-	^114^
		UHT	-	-	^114^
	Cefazolin	Raw	-	50	^101^^,^^125^
	Cephoperazone	Raw	-	50	^101^^,^^119^^,^^120^^,^^122^^,^^125^
	Dicloxacillin	Raw	-	30	^101^^,^^119^^,^^120^^,^^121^^,^^122^
	Ampicillin	Raw	-	4	^101^^,^^120^^,^^121^
	Cloxacillin	Raw	-	30	^101^^,^^134^^,^^120^^,^^121^
	Cefacetrile	Raw	-	125	^101^
	Chloramphenicol	Raw	-	-	^116^^,^^128^^,^^104^
	Rifaximin	Raw	-	-	^101^
Mycotoxins	Aflatoxin M _1_	Raw	0.5	0.05	^135^^,^^136^^,^^137^^,^^138^^,^^139^^,^^140^^,^^141^^,^^142^^,^^143^
		Pasteurized	-	-	^135^^,^^144^^,^^137^^,^^140^^,^^141^^,^^142^^,^^145^^,^^146^
		UHT	-	-	^137^^,^^141^^,^^147^^,^^148^^,^^145^^,^^146^
	Ochratoxin A	Raw	-	-	^135^^,^^136^^,^^137^
		Pasteurized	-	-	^135^^,^^144^^,^^136^^,^^149^
	α-zearalenol	Raw	-	-	^135^^,^^137^^,^^150^
		Pasteurized	-	-	^135^
	Fumonisin B1	Raw	-	-	^137^
		Pasteurized	-	-	^144^^,^^137^
	Fumonisin B2	Pasteurized	-	-	^144^
	β-zearalenol	Raw	-	-	^150^
		Pasteurized	-	-	^144^
	Zearalenone	Raw	-	-	^136^^,^^144^^,^^137^^,^^151^^,^^150^^,^^135^^,^^152^^,^^153^
		Pasteurized	-	-	^135^^,^^154^
	Aflatoxin B1	Raw	-	-	^136^^,^^137^^,^^140^
		Pasteurized	-	-	^144^^,^^136^^,^^137^^,^^140^
		UHT	-	-	^137^
	Aflatoxin B2	Raw	-	-	^137^
		Pasteurized	-	-	^137^
	Aflatoxin G1	Raw	-	-	^137^
		Pasteurized	-	-	^137^
	Aflatoxin G2	Raw	-	-	^137^
		Pasteurized	-	-	^137^
	Zearalanol	Raw	-	-	^137^
	α-zearalenone	Raw	-	-	^137^
	Cyclopiazonic acid	Pasteurized	-	-	^137^
	α-zearalanol	Pasteurized	-	-	^155^
	Deepoxy-deoxynivalenol	Raw	-	-	^137^
	Deoxynivalenol	Raw	-	-	^151^^,^^152^^,^^150^
	Aflatoxin M2	Raw	-	-	^138^
		Pasteurized	-	-	^137^
		UHT	-	-	^137^^,^^151^
Hormones	Leptin	Pasteurized	-	-	^124^^,^^156^
	Triiodothyronine and Thyroxine	Pasteurized	-	-	^156^
	Prednisolone	Raw	-	6	^124^^,^^138^
	Relaxin	Pasteurized	-	-	^156^
	Insulin	Raw	-	-	^157^
		Pasteurized	-	-	^156^^,^^157^
	Oxytocin	Pasteurized	-	-	^156^^,^^158^^,^^157^^,^^159^
	Adiponectin	Raw	-	-	^160^
		Pasteurized	-	-	^156^
	Estriol	Raw	-	-	^161^
		UHT	-	-	^160^^,^^162^
	17α-Estradiol	Raw	-	-	^161^
		Pasteurized	-	-	^162^
		UHT	-	-	^161^^,^^162^
	17β-Estradiol	Raw	-	-	^161^^,^^163^
		Pasteurized	-	-	^164^^,^^163^^,^^162^^,^^165^^,^^166^
		UHT	-	-	^161^^,^^162^
	Estrone	Raw	-	-	^161^^,^^167^^,^^163^
		Pasteurized	-	-	^164^^,^^163^^,^^162^
		UHT	-	-	^161^^,^^162^
	Testosterone	Raw	-	-	^161^^,^^167^
		Pasteurized	-	-	^161^
	4-Androstenediol	Raw	-	-	^161^
	5-Androstenediol	Raw	-	-	^161^
	4-Androstenedione	Raw	-	-	^161^
	Progesterone	Raw	-	-	^161^^,^^167^^,^^168^^,^^169^
		Pasteurized	-	-	^168^
		UHT	-	-	^161^
	17α-Hydroxyprogesterone	Raw	-	-	^161^
	Cortisone	Raw	-	-	^161^
	Cortisol	Raw	-	-	^161^^,^^170^
	Corticosterone	Raw	-	-	^161^
	Hydrocortisone	Pasteurized	-	-	^171^
	Insulin-like Growth factor-I	Raw	-	-	^157^
		Pasteurized	-	-	^58^^,^^157^^,^^171^
	Pregnenolone	Raw	-	-	^167^
	Androstenedione	Raw	-	-	^167^
		Pasteurized	-	-	^167^
	Dehydroepiandrostenedione	Raw	-	-	^167^
	5-α-Androstane-3,17-dione	Raw	-	-	^167^
	Prolactin	Pasteurized	-	-	^156^
	Growth Hormone	Pasteurized	-	-	^156^^,^^58^

MRLa: Maximum Residue Levels by Codex Alimentarius; MRLb: Maximum Residue Levels by European Union, EU. UHT: ultra-high temperature.

2.2.1 Pesticides

A variety of pesticide residues in detectable amounts in raw milk, pasteurized, and UHT (ultra-high temperature) milk has been reported by several authors. This is due, among other factors, to the lipophilic properties and resistance to biodegradation of these types of contaminants. ^8^ There are three possible forms in which pesticides can enter the animal's body ^172^: (i) through contaminated water, (ii) through the pores of the skin when the animal is sprayed or soaked to treat ectoparasites, and (iii) through contaminated feed and forage, the latter being the main source of entry.

(i) The presence of organophosphorus pesticide residues (malathion, methyl-parathion, diazinon, ethion) was identified. The average concentrations detected were 0.032-0.78, 0.13, 0.32-0.74, 0.010 μg/L for malathion, methyl-parathion, diazinon and ethion, respectively. ^62^^,^^173^ Fipronil and chlorpyrifos were other pesticides found in water samples supplied to livestock. ^174^^,^^175^ Ashoub & Azam ^176^ identified DDT (Dichloro diphenyl trichloroethane), aldrin, heptachlor epoxide, lindane, methoxychlor, diazinon, and deltamethrin in water samples from cattle farms. These same compounds have been identified in cattle drinking water and in cow's milk. ^11^^,^^47^^,^^51^^,^^54^^,^^55^^,^^62^^,^^65^^,^^177^^–^^180^ This verifies that water contaminated by pesticides and supplied to cattle is one of the main routes of contamination of raw cow's milk.
(ii) According to the analysis of Table 2, Claborn *et al*. ^181^ report the presence of malathion residues in cow's milk after cattle were sprayed with this pesticide for the treatment of ectoparasites. Malathion was found to be completely secreted from the udder 24 hours after application. In contrast to malathion, lindane was reported not to be completely excreted in milk until seven days after application to the cow's skin. ^182^ Residues of chlorpyrifos and ethion have been found in cow milk up to 24 and 72 hours after application, respectively. ^183^ This confirms that skin contaminated with these pesticides is another route of contamination of raw cow's milk.
(iii) In forage, concentrations of 0.02 mg kg ^-1^ of DDT residue were reported. ^184^ The presence of cypermethrin, chlorpyrifos, cyhalothrin, and deltamethrin in forage was reported in a range of mean concentrations between 1.03-6.01 ng g ^-1^. In addition to the presence of pesticides in forages, residues of lindane, DDT, fenvalerate, ethion, malathion, profenofos were also reported in feed. The mean concentrations of these varied in the range of 0.63-4.05 ng g ^-1^. ^175^ The presence of deltamethrin in feed was also reported in a concentration range of 41.99-381.30 μg kg ^-1^. ^185^ Another investigation revealed the presence of malathion, dimethoate, methyl-parathion, diazinon in the feed fed to cattle. The range of detected concentrations was between 0.01-80.45 μg L ^-1^. ^62^ All the contaminants reported in forage and feed were also detected in cow's milk. ^11^^,^^54^^,^^55^^,^^57^^,^^60^^–^^62^^,^^177^^,^^179^^,^^186^ Thus, like water, pesticide-contaminated forage and feed are a route of contamination as they are directly ingested by cattle and excreted through cow's milk.

Pesticides are one of the most commonly found contaminants, not only in raw cow's milk but also after the pasteurization and UHT process. Their presence in milk, even below the maximum permitted levels, represents a health risk to the consumer. It is related to Hodgkin's disease (HD), non-Hodgkin's lymphoma (NHL), Parkinson's disease, endocrine disruption, respiratory and reproductive disorders, among others. ^187^

It is important to note that organochlorine pesticides such as hexachlorocyclohexane, dichloro diphenyl trichloroethane, and endosulfane are still present despite having been banned since the 1970s because of their high persistence in the environment and their harmful effects on human health, ^188^ are still detected in cow's milk. This indicates that they are still used in agriculture and animal husbandry. With a few exceptions (cyhalothrin, cypermethrin, fenvalerate, deltamethrin, permethrin, and diazinonella), the vast majority of pesticides found in cow's milk are not regulated by Codex and the EU. This demonstrates the low efficiency of the regulatory controls of these contaminants in the unprocessed and post-processed product, leading to an inefficient safety of this food product.

2.2.2 Metals

Although metals are found in the environment either naturally or due to industrial and/or agricultural activities, there are several routes by which they reach the milk. Namely, ingestion of contaminated food, fodder, and/or contaminated drinking water. In the soil, they are absorbed by many crop plant species, which, when ingested by animals, are transferred to the lactating glands and finally excreted in milk. ^172^ Equipment used in the dairy industry is another source of contamination directly to milk with metals such as chromium and nickel. ^189^ Heavy metals such as cadmium, lead, mercury, and arsenic reach milk by indirect contact through feed consumed by cattle. ^189^ Although the literature does not report the presence of metals in water or fodder destined for cattle, as well as in pesticides, these can be another of the main routes of contamination.

Several heavy metals have been reported in the literature to be found in raw cow's milk. The metals least found in studies of raw cow milk are tin and molybdenum. These elements are not abundant in nature, and their presence in fodder or water for animal consumption will depend on soil characteristics, while the most reported are lead, cadmium, copper, and zinc, due to environmental pollution produced by man mainly in industrial activities. ^79^^,^^190^ Minerals such as Fe, Cu, and Zn are necessary for various biological functions. However, high concentrations of these minerals have negative effects on human health. ^96^ Lead is one of the non-essential metals classified as carcinogenic to humans by the International Agency for Research on Cancer. ^191^ Cadmium is associated with the formation of human lung, kidney, breast, prostate, urinary tract cancer because it affects cell proliferation, differentiation, and other cellular activities. ^192^

None of the heavy metals reported in the literature consulted have established maximum residue limits (MRLs) by Codex ^193^ and the EU. ^194^ However, these contaminants are known to represent a high risk to human health. Stricter control measures should be adopted in the dairy industry, considering that cow's milk is one of the most consumed products by humans worldwide.

2.2.3 Antibiotics

Antibiotics are used in livestock activities in three basic ways: therapeutic, prophylactic, and growth promoters. About 80% of dairy cattle are subjected to antibiotic treatments on at least one occasion throughout their lives, mostly used as growth promoters and for the treatment of various diseases such as mastitis, arthritis, respiratory diseases, gastrointestinal diseases, and bacterial infections. ^195^ Cows eliminate antibiotics and their metabolites through milk, depending on the dose and route of application, level of milk production, type and degree of mammary disease, and time between treatment and milking. On the other hand, oral, intramuscular, or intravenous administration is less important from the point of view of milk hygiene than intramammary application. However, intramammary antibiotics are easy to apply and generally cheaper, so they are preferred in dairy farms.

The most common disease in dairy cows is mastitis, whose treatment includes the wide use of tetracyclines, β-lactams, oxytetracycline, difloxacin, among others, being the β-lactams of greater application. ^8^ Within the latter group, the most employed are penicillin, ampicillin, and amoxicillin. ^196^ According to the literature, the presence of antibiotics in milk has been evidenced, highlighting tetracycline, oxytetracycline, penicillin, and amoxicillin. ^103^^,^^124^^,^^197^^,^^198^ While other antibiotics less reported in milk were rifamixin, gatifloxacin, spiramycin, and lomeflaxacin, with no indication in the studies of the purpose of their application in cattle. ^101^^,^^112^^,^^126^^,^^127^

The consumption of contaminated milk with antibiotic residues is an emerging public health problem worldwide. Therefore, it is important to control the presence of antibiotic residues in food to avoid the appearance of resistance to these antibiotics in humans. The presence of antibiotics at concentrations even below the MRL in milk can cause undesirable effects on human health such as ototoxicity and nephrotoxicity, ^199^ endocrine disruption, ^200^ hypersensitivity, and especially bacterial resistance. ^130^ According to the literature consulted, 43 antibiotics present in cow's milk have been identified, of which 18 are not regulated by Codex ^193^ and EU standards. ^194^

Considering that the use of antibiotics in cattle generates residues in milk, their excessive use should be avoided, and the elimination times before milking should be respected in order to avoid the presence of these contaminants.

2.2.4 Mycotoxins

The quality of food products is commonly affected by toxin contamination, of which 60 to 80 % are caused by mycotoxins. ^201^ This means a risk for human health and great economic losses in the industrial sector.

Mycotoxins are natural contaminants produced by *Aspergillus*, *Penicillium,* and *Fusarium* fungi, ^154^ the most prominent being AFM1, which results from the metabolism of aflatoxin B1 in the liver of contaminated animals. ^15^^,^^143^ In the 1960s, the first reported case of aflatoxin contamination was reported for the first time, beginning the concern for this type of contaminant. Even during this decade, high consumption of feed contaminated by this mycotoxin was reported, which led to indirect contamination of cow's milk for consumption, compromising the safety of this product. ^202^ Therefore, it is considered that the main routes of entry of mycotoxins into milk are contaminated crops and feed ingested by cows. ^136^

It is known that approximately 0.3-6.2% of AFB1 (Aflatoxin B1) present in animal feed is converted to AFM1. ^15^ This mycotoxin is neither degraded nor removed by industrial food processes such as pasteurization and sterilization, nor by the cooking of feed. ^203^ This represents a difficult problem to deal with at the industrial level due to the stability of mycotoxins in general to thermal, physical, and chemical treatments. ^204^

AFM1 mycotoxin is the only regulated by Codex ^193^ and EU ^194^ and the most reported in cow's milk according to the literature. However, other abundant mycotoxins have been identified in this food product, such as ochratoxin A and zearalenone. The fungi of the genus *Aspergillus* and *Penicillium* produce Ochratoxin A, while fungi of the genus *Fusarium* produces zearalenone, commonly found in cattle feed. ^138^ On the other hand, aflatoxin G2, aflatoxin G1, aflatoxin B2, and zearalanol show a lower incidence in cow's milk. The literature on the effects on human health associated with the ingestion of mycotoxin-contaminated milk is scarce or almost non-existent, unlike AFM1. Therefore, studies on this type of contaminants should be expanded.

2.2.5 Hormones

The use of hormones in the livestock industry increases production yields and medical treatments. Their fat-soluble characteristics favor their high persistence and presence in cow's milk due to the high-fat contents. ^156^ Therefore, the supply of hormones to cattle represents a form of direct contamination that, like other contaminants, is excreted through milk. However, the European Union banned the use of hormones through the Directive 96/22/EC, and enforcement is regulated by Directive 96/23/EC. ^165^

Prednisolone in combination with amoxicillin and clavulanic acid is used to treat mastitis in cows' udders, ^205^ being an access route of this contaminant to milk. The 17β-estradiol and progesterone, with the highest presence in cow milk, are sex hormones widely used to induce lactation, improve fertility and synchronize the estrous cycle. ^8^^,^^168^ The hormones least found in studies in milk were testosterone, somatostatin, and cortisone. The presence of estrogens in cow's milk has been linked to diseases such as breast cancer ^206^ and conditions in the gastrointestinal tract. ^156^ Other diseases associated with the presence of hormones in cow's milk have included acne, prostate cancer, uterine cancer, and male reproductive disorders. ^167^

Table 2 shows that several hormones are frequently present in cow's milk, with prednisolone being the only one regulated by the EU. ^194^ This indicates that regulations should be established for different hormones considering that they are the chemical compounds mostly used to increase milk production yield to preserve quality and consumer safety.

## 3. Pasteurization process in cow's milk

The principles and name of pasteurization come from the studies of the French scientist Louis Pasteur. His interest in milk and other food products was due to their putrefaction, which he later attributed to the growth of undesirable microorganisms. ^207^ Several pathogenic microorganisms are found in raw milk: *Pseudomonas*, *Enterobacter*, *Bacillus*, *Clostridium*, *Microbacterium,* and *Micrococcus.* Pathogenic microorganisms in cow's milk have been linked to infectious diseases such as campylobacteriosis, salmonellosis, yersiniosis, listeriosis, tuberculosis, brucellosis, staphylococcal enterotoxin intoxication, streptococcal infections, and *Escherichia coli* O157: H7 infection. ^208^

It was not until the end of the 1880s that heat treatment began to be used to commercialize milk. This arose with the main objective of inactivating *Mycobacterium tuberculosis*, the cause of tuberculosis in humans associated with the consumption of raw milk. Thus, pasteurization became a process universally employed by developed countries after World War II. However, there is evidence that not all pathogenic microorganisms can be eliminated during pasteurization, such as *Staphylococcus aureus*, *micrococci*, *Streptococcus spp,* and *Bacillus.* ^209^ Which calls into question the efficiency of this process.

The US Food and Drug Administration (FDA) establishes a maximum limit for bacteria in raw cow's milk of 100,000 cfu ml ^-1^ and 20,000 cfu ml ^-1^ for pasteurized milk. ^209^

Pasteurization is a technology classified on the basis of operating temperatures and exposure times as follows: LTLT, HTST, and UHT. Low-temperature long-time pasteurization (LTLT) uses a minimum temperature of 62.8°C and a minimum time of 30 min. High-temperature short-time pasteurization (HTST) uses a minimum temperature of 71.1°C, a minimum time of 15 seconds, and ultra-high temperature pasteurization (UHT) works at a minimum of 135°C and during a minimum time of 1 second. ^210^ Pasteurized milk under UHT conditions can be stored for several months without refrigeration. ^211^ Whereas the shelf life of pasteurized milk ranges from 10 to 20 days when kept under refrigerated conditions below 6.1°C. ^212^

It has been shown that the application of pasteurization denatures proteins with bacteriostatic capacity, as is the case of lactoferrin. This is a glycoprotein that binds iron, and its complete denaturation has been evidenced losing its inhibitory capacity on *Escherichia coli* under UHT conditions. ^213^ For this reason, it is suggested that heat treatment should be applied below 75°C to avoid denaturation of proteins with bacteriostatic capacity and at the same time cause inactivation of pathogenic microorganisms. ^213^

On the other hand, the HTST process degrades up to 20% of the vitamins (B1, B6, B12, and C) present in milk. ^214^ This evidence shows that, although pasteurization and UHT have been widely used to eliminate pathogenic microorganisms, it is not entirely efficient for this purpose. There are even losses of milk mineralization, varying its nutritional composition.

The presence of microbial contaminants in different samples of pasteurized milk shows that, although pasteurization aims to eliminate microorganisms present in milk, it is not totally effective. Moreover, with the appearance of other contaminants, the quality of milk no longer depends only on the presence of microorganisms. It is, therefore, necessary to study other methods of decontamination to ensure the safety and health of consumers.

## 4. Alternative methods for the treatment of cow's milk

International regulations require maximum limits for microbial and chemical contaminants to ensure the quality of drinking milk. Pasteurization is a technology widely used in the dairy industry. However, it is exclusive for the elimination of microbial contaminants. The literature mentions alternatives for eliminating specific microbial and chemical contaminants ( Table 3).

**Table 3.** Alternative methods to pasteurization for removal of contaminants in bovine milk.

	Contaminant	Process	Reference
Pathogens	*Escherichia coli*	Inactivation with supercritical carbon dioxide technology	^215^
	*Escherichia coli* and *Listeria innocua*	Inactivation using a UV-C thin film reactor	^216^
		Inactivation by pulsed electric fields	^217^
	Aerobic bacteria	Reduction by UV-C irradiation	^218^
	*Listeria monocytogenes*	Inactivation by ozonation	^219^
	*Staphylococcus aureus*, *Listeria monocytogenes*, *Lactobacillus plantarum*, *Lactobacillus pentosus, Salmonella Typhimurium, Escherichia coli, Pseudomonas fluorescens*	Inactivation by combinations of ultrasound, hydrogen peroxide, and active lactoperoxidase system	^220^
	Aerobic bacteria, coliforms, yeasts, and molds	Inactivation by carbon dioxide at high pressure	^221^
	*Escherichia coli, Salmonella, Listeria monocytogenes, Enterobacteriaceae, lactic acid bacteria, and Pseudomonas* spp.	Inactivation by high-pressure processing	^222^
	*Escherichia coli,* Salmonella, yeasts, and *lactobacillus* spp.	Inactivation by ND-YAG laser	^223^
	*Pseudomonas aeruginosa, Escherichia coli, Staphylococcus aureus,* and *Listeria innocua*	Inactivation by pulsed electric fields	^224^
	*Escherichia coli, Staphylococcus aureus,* and *Pseudomonas fluorescens*	Inactivation by manothermosonication	^225^
Pesticides	Organophosphates (chlorpyrifos, diazinon, fenitrothion, malathion, methyl parathion)	Degradation by lactic acid bacteria	^226^
	Methyl parathion	High-intensity ultrasound	^227^
	Dimethoate, fenthion, malathion, methyl parathion, monocrotophos, phorate, and trichlorfon	Degradation by *lactobacillus* spp. bacteria at 42°C	^228^
Metals	Pb ^2+^ and Hg ^2+^	Adsorption with pluronic p123 diacrylate hydrogels	^229^
	Lead	Biosorption with *Saccharomyces cerevisiae*	^230^
		Biosorption with *Lactobacillus acidophilus* ATCC 4356	^231^
	Mercury	Biosorption with *Lactobacillus acidophilus* ATCC 4356	^232^
		Biosorption with *Saccharomyces cerevisiae*	^233^
	Copper	Adsorption using imac hp resin	^92^
	Cadmium	Biosorption with *Saccharomyces cerevisiae*	^234^^,^^235^
		Biosorption with *Lactobacillus acidophilus* ATCC 4356	^231^
Antibiotics	Amoxicillin, doxycycline, ciprofloxacin, and sulfadiazine	Ozonization	^236^
	Chlortetracycline and cefazolin	Electrochemical method	^237^
	Tetracycline	Electrochemical method	^238^
		Adsorption with molecularly imprinted polymer	^239^
	Ciprofloxacin	Adsorption with BiPO _4_ @ fluorescent photocatalytic graphene oxide-based magnetic molecular imprinted polymer	^240^
	Amoxicillin, ciprofloxacin, doxycycline	Decomposition by gamma irradiation	^241^
Mycotoxins	Aflatoxin M1	Adsorption with molecularly imprinted polymer coated on the surface of the stainless-steel plate	^242^
		Removal using *Saccharomyces cerevisiae* and *Lactobacillus helveticus*	^243^
		Adsorption with clay minerals (kaolin and bentonite)	^244^
		Elimination by a combination of yeast and probiotic bacteria species	^245^^,^^246^
		Biofilm elimination of *Lactobacillus rhamnosus gg*	^247^
		Adsorption with clay minerals (kaolin and bentonite)	^248^

UV-C: Ultraviolet-C (200-280 nm); Nd:YAG: neodymium-doped yttrium aluminum garnet; Pb: lead; Hg: mercury; ATCC: American Type Culture Collection; BiPO _4_: Bismuth phosphate (III).

Supercritical carbon dioxide has been used as an inactivating agent for *E. coli*, where the greatest reduction in the content of microorganisms was observed during a residence time of 20 minutes, achieving almost complete inactivation after 70 minutes. ^215^ Complete inactivation of coliforms, molds, and yeasts was achieved, while a maximum reduction of aerobic bacteria of 4.96 log was obtained using high-pressure carbon dioxide. ^221^ Using a thin-film UV-C (Ultraviolet-C) reactor with flow-guiding elements allowed a 4.58 log and 3.19 log reduction for *E. coli* and *L. innocua*, respectively. ^216^ Makarapong *et al*. ^218^ employed a UV-C reactor for the inactivation of aerobic bacteria achieving a 4.60 log and 4.70 log reduction at 48W and 39W, respectively. UV-C lamp wattage did not significantly influence the fat concentration in the milk, which means that it is necessary to improve the method to guarantee an effective reduction of these microorganisms if milk transport time exceeds two hours without cooling. It was verified that *L. monocytogenes* was completely inactivated in milk with ozone for 15 minutes. However, nutritional values were affected. ^219^ Exposure of milk to Nd:YAG laser did not alter the physicochemical properties of milk, but the percentage of reduction was low for *E.coli* (30%), *Salmonella* sp (25%), yeasts (47%), and *Lactobacillus* sp (30%). ^223^ The combination of ultrasound with hydrogen peroxide and an active lactoperoxidase system was able to guarantee the microbial quality of milk as it was able to completely inactivate *Staphylococcus aureus*, *Listeria monocytogenes*, *Lactobacillus plantarum*, *Lactobacillus pentosus*, *Salmonella Typhimurium*, *Escherichia coli,* and *Pseudomonas fluorescens* at 10 minutes at an amplitude of 125 μm. ^220^ The application of ultrasound in combination with variations in temperature, time, and constant pressure (manothermosonication) achieved minimal reductions of up to 1.6 log CFU/ml for *E. coli* and *P.fluorescens* and 1.05 log CFU/ml for *S. aureus.* Further studies are needed to ensure effective inactivation using manothermosonication. ^225^ The application of high pressures (400-600MPa) effectively inactivated (5 log CFU/ml) *E. coli*, *Salmonella* and *L. monocytogenes*, *Enterobacteriaceae*, lactic acid bacteria, and *Pseudomonas* spp. ^222^ One of the most widely used methods for the inactivation of microorganisms in cow's milk is pulsed electric fields (PEF). This method was applied for the inactivation of *E.coli* and *L. innocua*, achieving a reduction of 2 log CFU/ml. ^217^ It was found that combining this method with preheating at 50°C achieved a 5-6 log CFU/ml reduction of *Pseudomonas aeruginosa* and a total reduction of *E. coli*, *S. aureus*, and *L. innocua.* ^224^

Biosorption methods employing the use of microorganisms prove to be efficient in the removal of pesticides, metals, and mycotoxins. Biosorption with lactic acid bacteria managed to eliminate organophosphate pesticides from cow's milk, being more effective for chlorpyrifos, fenitrothion, and malathion, whose degradation constants were greater than 0.018 h ^-1^. On the other hand, diazinon and methyl parathion were more resistant when applying of the different strains of lactic acid bacteria separately and in combination. The degradation rate constants were correlated with the measurement of phosphatase activity, and it was found that the lower the phosphatase activity, the lower the degradation constant. ^226^ The same method was applied for this group of contaminants finding that dimethoate and methyl parathion were the most stable with the lowest degradation rate constants (0.0165-0.0184 and 0.0213 h ^-1^, being more efficient for the removal of malathion with higher degradation rate constants (0.0218-0.0420 h ^-1^). ^228^ Although the application of lactic acid bacteria was shown to be an effective method for removing diazinon, dimethoate, and methyl parathion in cow's milk it was not very selective since it cannot eliminate all the organophosphates studied.

Biosorption with *Saccharomyces Cerevisiae* allowed the removal of 70% of lead, mercury, and cadmium metals. ^230^^,^^233^^–^^235^ The removal percentage was higher when *Lactobacillus Acidophilus* was used, eliminating 80, 75, and 72%, respectively. ^231^^,^^232^ The use of *Saccharomyces cerevisiae* and *Lactobacillus helveticus* removed AFM1 from milk by an as yet unknown binding mechanism. ^243^ A combination of probiotic bacteria with yeast species managed to remove 90.88% of AFM1 within 72 hours. ^245^ This percentage of removal was higher than that obtained in another study (19-61%). ^246^ By applying a biofilm of *Lactobacillus rhamnosus*, an AFM1 removal of 60.74% was achieved. Despite that, the method is not a viable alternative for application because a reduction in the percentage of fat and total dry matter was observed. ^247^

Biosorption methods employing microorganisms ( *Lactobacillus acidophilus* and *Saccharomyces cerevisiae*) are efficient for removing heavy metals in cow's milk (lead, mercury, copper, and cadmium). However, they require a minimum fermentation period of 4 days. When using lactic acid bacteria to degrade organophosphorus pesticides, a minimum fermentation period of 24 hours is required. These times would represent economic losses for the industry, and given the existing world demand for milk, it would be almost impossible to apply them on a large scale.

Adsorption methods prove to be efficient for removing metals, antibiotics, and mycotoxins. By adsorption with diacrylate Pluronic P123 (P123-DA) hydrogels removed about 85.3% and 81.9% of Pb ^2+^, and Hg ^2+^ ions, respectively. ^229^ Resins have been another adsorbent used in the adsorption of heavy metals in cow's milk. IMAC HP resin was described for the removal of copper ions (76.89%). ^92^ Tetracycline, oxytetracycline, chlortetracycline, and doxycycline have been removed by adsorption on a molecularly imprinted polymer, achieving 81.83, 95.47, 96.44, and 93.25% removal, respectively. ^239^ A photocatalytic-fluorescent polymer, produced from graphene oxide and bismuth phosphate with molecular magnetic imprinting, allowed ciprofloxacin's complete degradation. ^240^ Bodbodak *et al*., ^242^ developed a molecularly imprinted polymer coated on the surface of a stainless-steel plate as an adsorbent material for the decontamination of AFM1 in cow's milk. This method was able to remove 87.3 to 96.2% of AFM1 without causing a change in the physicochemical properties of the milk. Adsorption with kaolin and natural calcium bentonite clay for adsorption was able to remove AFM1 by 86.1-93.3% and 93.7-97.7%, respectively. It was observed that no change in the nutritional properties of milk would occur. ^244^ Despite this, few studies have been reported in cow's milk. Therefore, there are not enough to consider its application at the industrial level.

Other methods less reported in the literature were also applied for the removal of pesticides and antibiotics. The ultrasonic treatment proved to be effective for the degradation of 97.10% of methyl parathion. However, this method is limited by the generation of degradation products with toxic effects. ^227^ For the elimination of antibiotics in cow's milk, methods such as ozonation have been applied, with about 95% degradation for amoxicillin, doxycycline, ciprofloxacin, and sulfadiazine. ^236^ Electrochemical oxidation applied for the removal of small concentrations of chlortetracycline, cefazolin, ^237^ and oxytetracycline ^238^ was also described. Gamma radiation was also found to be effective for the removal of amoxicillin, ciprofloxacin, and doxycycline by 90% in cow's milk samples. ^241^ However, of all the antibiotics detected in cow's milk, they have only been tested for the elimination of amoxicillin, doxycycline, ciprofloxacin, sulfadiazine, chlortetracycline, cefazolin, y tetracycline. More studies are needed to validate the application of these methods for the decontamination of cow's milk.

It has not been demonstrated that a single method is capable of eliminating different groups of contaminants, as is the case of pasteurization for microbial contaminants. Despite the wide use of hormones in the cattle industry and their consequent generation of traces in cow's milk, no removal methods have been reported for them. The alternative methods studied to date have been applied on an industrial scale, and many of them alter the nutritional properties of milk. The fact that most of these chemical contaminants are not regulated by standards does not oblige the dairy industry to use alternative methods to pasteurization. Nor is it economically viable to use a different method for the elimination of each contaminant present in milk. However, to guarantee the safety of milk, it is essential to study processes that complement pasteurization and can eliminate pathogenic microorganisms and chemical contaminants.

## 5. Conclusions and future prospects

The presence of contaminants in raw cow's milk (many of them banned) is an indication that they are currently used illegally in both agriculture and animal husbandry. Although the presence of contaminant residues in milk represents a health risk to the consumer, there are no MRLs established for all of them. In addition, pasteurization processes are not efficient for the degradation or elimination of the different contaminants addressed.

Although, the literature exposes alternative methods for removing various contaminants in milk, they are still not sufficient nor applied on an industrial scale. Instead, they have been applied individually or in very small families of contaminants. There are no evidence or results concerning the interactions between them or with intermediate products formed on cow's milk, nor changes in the organoleptic properties. A particular case is hormones, which although they are a direct source of contamination, with evidence of their presence in raw, pasteurized, and UHT milk, the literature does not report specific elimination methods for these types of contaminants.

However, alternative methods have proven to be efficient in degrading several contaminants present in milk. Based on this hypothesis, it is suggested to deepen the application of these methods, including the study of interactions between different families of contaminants, application of new materials, or modification of existing ones. Studies on toxicity or changes in organoleptic properties. In this sense, the field of nano-biotechnology, nano-fibers, nano-membranes, biochar, MOF's (metal-organic framework), among others, could play a relevant role, guaranteeing the safety of the milk consumed, and consequently, a better quality of life for consumers.

## Data availability

No data are associated with this article.
