# Supplementary material for: Contaminants in the cow's milk we consume? Pasteurization and other technologies in the elimination of contaminants
Source: F1000Res. 2022 Jan 25;11:91. [Version 1] doi: 10.12688/f1000research.108779.1 (PMC8822143; doi:10.12688/f1000research.108779.1)
Supplement: Peer review history

# Peer review: Reviewer response for version 1

**DOI:** 10.5256/f1000research.120201.r121010

**Published:** 2022-02-09

**Authors:** Lourdes Casas-Cardoso

**Affiliations:** 1 Department of Chemical Engineering and Food Technology, Faculty of Science, International Campus of Excellence in Agrifood, ceiA3, University of Cadiz, Puerto Real, Spain

The review topic: "Contaminants in cow's milk, pasteurization and alternative technologies in the removal of these contaminants" is discussed extensively in the context of the paper.

 Table 1 shows the pathogens in cow's milk reported in the literature and Table 2 summarizes the chemical contaminants in cow's milk reported in the literature. All statements are adequately supported by citations.

 Pasteurization is a technology used in the industry, however it is exclusive for the elimination of microbial contaminants. Table 3 describes alternative methods to pasteurization: supercritical carbon dioxide, inactivation by pulsed electric fields, inactivation by ozonation. Alternative methods have proven to be efficient in degrading several contaminants; however, they are still not sufficient nor applied on an industrial scale.

 The conclusions are appropriate. It is suggested to continue research on alternative methods.

Is the review written in accessible language?

Yes

Are all factual statements correct and adequately supported by citations?

Yes

Are the conclusions drawn appropriate in the context of the current research literature?

Yes

Is the topic of the review discussed comprehensively in the context of the current literature?

Yes

Reviewer Expertise:

natural products, supercritical fluid extraction, countercurrent extraction column, supercritical impregnation, active compounds

I confirm that I have read this submission and believe that I have an appropriate level of expertise to confirm that it is of an acceptable scientific standard.

# Peer review: Reviewer response for version 1

**DOI:** 10.5256/f1000research.120201.r121007

**Published:** 2022-02-09

**Authors:** Qiansheng Huang

**Affiliations:** 1 Center for Excellence in Regional Atmospheric Environment, Key Lab of Urban Environment and Health, Institute of Urban Environment, Chinese Academy of Sciences, Xiamen, China

**This review attempts to summarize the knowledge generated with respect to the various types of contamination that cow's milk has and the effect they have on human health. As a central axis, it focuses on strategies to eliminate these contaminants so that their consumption is safer over the population in general. The treated theme is significant, with a real involvement in food security. I consider that the manuscript deserves to be published, not before taking into account the comments detailed below:**

**COMMENT 1:** This idea "they have not demonstrated efficacy in elimination contaminants" is repetitive in the summary.

**COMMENT 2:** The first paragraph of the introduction is not really a paragraph but a single long sentence. Please correct those errors throughout the document for readability.

**COMMENT 3:** Second paragraph of the introduction, last line: please be specific when "United States" e.g. United States of America.

**COMMENT 4:** The last sentence of the first paragraph of section 2, this must be better organized, the phrase is very long and can lead to confusion. Additionally, use the punctuation signs correctly.

**COMMENT 5:** In the fourth paragraph of section 1, reference is made to pasteurization is a method considered for the elimination of "non-chemical compounds". Specify what types and metabolites resulting from the application of this process.

**COMMENT 6:** The following phrase, "For better analysis and understanding, the classification of contaminants according to the origin is microbial contaminants and chemical contaminants (Figure 2)" must be incorporated into a paragraph, it is not correct to leave it alone.

**COMMENT 7:** In the paragraph before point 2.1, two contiguous phrases initiate in this same way: "Among chemical contaminants.....". Please correct that.

**COMMENT 8:** This phrase "The main types of microorganisms present in milk are bacteria, yeasts, and molds, which represent the different types of microorganisms present in cow's milk." It is redundant. Please improve it.

**COMMENT 9:** When a bibliographic reference is made within the text, indicate the year after appointing the authors. Apply in all cases.

**COMMENT 10:** In reference to Figure 1: specify contaminants that come from milk containers (each type).

**COMMENT 11:** Last paragraph of section "2.", third line: indicate the reasons why there may be remanence of microbes even after pasteurization, considering that the process (pasteurization) has been carried out correctly.

**COMMENT 12:** SECTION 2.1: Here the "non-pathogenic" organisms should be included and those that cause alteration of milk, whose result is harmful to consumer.

 Improve the format of the tables.

**COMMENT 13:** SECTION 2.2.1, Literal "ii". What mean "According to the analysis of Table 2, Claborn et al.181 report……". What type of analysis was made with "Table 2"?, Who made it?

**COMMENT 14:** SECTION 2.2.1, Literal "II". In reference to "This confirms that skin contaminated with these pesticides is another route of contamination of raw cow's milk." The evidence presented is causal, the most appropriate term, in this case, would be "evidence suggests...". "

**COMMENT 15:** SECTION 2.2.1, Literal "II". "In forage..." is indicated. Specify the conditions of the sample and the possible source of contamination, allowing each case to understand more dynamically. Apply in all cases.

**COMMENT 16:** Throughout the text, there are many redundant ideas in the same phrase or paragraph, please correct that.

**COMMENT 17:** SECTION 2.2.1: Include contaminants such as PFAAs and other organic compounds.

**COMMENT 18:** SECTION 2.2.2: Is not informative and redundant in front of other sections of the document. Improve it by including figures or tables with broader data.

**COMMENT 19:** Linking words and phrases like "such as...." are used excessively. Please vary the expressions used in the text. Apply in all cases.

**COMMENT 20:** SECTION 2.2.3, first sentence. Be more specific in the statement.

**COMMENT 21:** SECTION 2.2.3, Second paragraph, first sentence. Here is specified about mastitis, although this pathology has already been named previously. Any specification of some pathology must be made it the first time in which it is named. A similar case occurs with the pasteurization reference on page 16.

**COMMENT 22:** The document has good information, but this is shown a little messy. The writing and sequential logical structure of the manuscript are the main problems. Also, it is necessary to improve the format of the tables. Improve the resolution of Figure 2.

**COMMENT 23:** Specify the harmful effects of each type of contaminant. Specify the accumulation of every contaminant depending on the milk class (which type of cow produces). Specify the difference, in relation to the presence of contaminants, according to the fat content of the milk (whole, half- skimmed, and skimmed).

**COMMENT 24:** SECTION 2.2.5: Include the explanation about the hormones of the cows depending on the life stage of them, and its effect on the consumer.

**COMMENT 25:** SECTION 3: It is messy, it does not have a "friendly" order for the reader. Address the actual effect that the pasteurization process has on the structure of each type of contaminant (chemical contaminants).

**COMMENT 26:** TABLE 3: Describe the metabolites produced by alternative methods for the elimination of chemical contaminants.

**COMMENT 27:** Describe the effect of each type of contaminant removal method on the nutritional profile of milk.

Is the review written in accessible language?

Yes

Are all factual statements correct and adequately supported by citations?

Yes

Are the conclusions drawn appropriate in the context of the current research literature?

Yes

Is the topic of the review discussed comprehensively in the context of the current literature?

Yes

Reviewer Expertise:

public health

I confirm that I have read this submission and believe that I have an appropriate level of expertise to confirm that it is of an acceptable scientific standard, however I have significant reservations, as outlined above.

# Peer review: Reviewer response for version 1

**DOI:** 10.5256/f1000research.120201.r121006

**Published:** 2022-02-07

**Authors:** Esmeralda García Díaz

**Affiliations:** 1 Centro de Química, Instituto de Ciencias, Benemérita Universidad Autónoma de Puebla, Puebla, Mexico

The manuscript entitled: "Contaminants in the cow's milk we consume? Pasteurization and other technologies in the elimination of contaminants" is valuable and presents an exhaustive review of the different types of contaminants in raw/pasteurized cow's milk and analyze the application of alternative processes for the elimination or degradation of contaminants. It provides relevant information about the sources of contamination and the health implications of ingesting these contaminants through milk, adequately supported by citations. It presents a wide variety of contaminants with their details regarding the contamination of milk. Information about alternative treatments to remove contaminants is also relevant and abundant. However, the tables need to be improved to present relevant information, which the authors can surely provide. Please see the following comments regarding the tables:

**COMMENTS**

 in Figure 1, the "processing and packaging stages" label is missing. Plasticizers that are used in containers, such as BPA need to be included.

 Robert Frankowski, Tomasz Grześkowiak, Beata Czarczyńska-Goślińska & Agnieszka Zgoła-Grześkowiak (2022) Occurrence and dietary risk of bisphenols and parabens in raw and processed cow’s milk, Food Additives & Contaminants: Part A, 39:1, 116-129, DOI: 10.1080/19440049.2021.1986234

 A better title for figure 2 would be "Incidence of contaminants in bovine...

 Page 4, sentence “For better analysis and understanding, the classification of contaminants according to the origin is microbial contaminants and chemical contaminants (Figure 2)”, needs to be rewritten, since the word "contaminants" is repeated several times. I suggest: Figure 2 presents the classification of cow´s milk contaminants and their microbial or chemical origin.

 Table 1 is long but contains relatively little information for its size. The column “Type of milk” is repetitive - separate horizontally by type of milk and add a column with the main identification method used in each case.

 Table 2 is also long and contain repetitive information, with columns labelled as MRL being practically empty.  it would be more useful to put the concentration interval reported in the referred works. MRL data can be mentioned in text.

 Throughout the document, round percentage values to make analysis easy.

 Page 14,

 In the sentence “Therefore, it is important to control the presence of antibiotic residues in food to avoid the appearance of resistance to these antibiotics in humans”.

 Who becomes resistant, humans or microorganisms? Its unclear in the sentence with the expression “appearance of resistance”.

 In sentence “Considering that the use of antibiotics in cattle generates residues in milk, their excessive use should be avoided, and the elimination times before milking should be respected in order to avoid the presence of these contaminants”.

 Please, reference the “elimination times before milking” to support the establishment of this time in some reported work.

 Page 16

 The sentence “The literature mentions alternatives for eliminating specific microbial and chemical contaminants”, change 'mentions' to 'reports' instead.

 This sentence is confusing:  “UV-C lamp wattage did not significantly influence the fat concentration in the milk, which means that it is necessary to improve the method to guarantee an effective reduction of these microorganisms if milk transport time exceeds two hours without cooling”.

 It’s not clear the relationship between lamp wattage, fat concentration, microorganisms and time of cooling.

 My conclusion: accept after minor revision.

Is the review written in accessible language?

Yes

Are all factual statements correct and adequately supported by citations?

Yes

Are the conclusions drawn appropriate in the context of the current research literature?

Yes

Is the topic of the review discussed comprehensively in the context of the current literature?

Yes

Reviewer Expertise:

Adsorption and photocatalysis of emerging pollutants of water. Chromatography of compounds of ambiental and biological interest

I confirm that I have read this submission and believe that I have an appropriate level of expertise to confirm that it is of an acceptable scientific standard.

## References

[1] : Occurrence and dietary risk of bisphenols and parabens in raw and processed cow's milk. *Food Addit Contam Part A Chem Anal Control Expo Risk Assess* .2022;39(1) : 10.1080/19440049.2021.1986234 116-129 10.1080/19440049.2021.1986234 34702142

# Peer review: Reviewer response for version 1

**DOI:** 10.5256/f1000research.120201.r121005

**Published:** 2022-02-02

**Authors:** Pawel Konieczynski

**Affiliations:** 1 Department of Analytical Chemistry, Medical University of Gdansk, Gdansk, Poland

In my opinion the article entitled: "Contaminants in the cow's milk we consume? Pasteurization and other technologies in the elimination of contaminants" is valuable and covers an important problem from the point of view of studies of food safety, especially milk. It was written based on wide literature screening (about 250 references) and presents the data and conclusions in a clear and comprehensive way. However, I have two comments:

 1. Why didn't the authors discuss the contaminants in milk (metals especially) in the context of norms of PTWI or ADI?

 2. Please add "Metals and metalloids" in the heading of Table 2 (page 8) since selenium is included in it.

 My conclusion: accept after minor revision

Is the review written in accessible language?

Yes

Are all factual statements correct and adequately supported by citations?

Yes

Are the conclusions drawn appropriate in the context of the current research literature?

Yes

Is the topic of the review discussed comprehensively in the context of the current literature?

Yes

Reviewer Expertise:

analytical chemistry

I confirm that I have read this submission and believe that I have an appropriate level of expertise to confirm that it is of an acceptable scientific standard.
